# The Uses of Forests

**Published:** 1888-05

**Authors:** 


### THE USES OF FORESTS.

Some time back the world was of opinion that trees were of value merely as supplies of timber, and that where building materials could be easily imported a country might, without any disadvantage, be laid entirely bare. To be sure, a few far-seeing individuals, such as Bernard Palissy, were aware of the influence of woodlands as regulators of climate. Similar views were taken in antiquity by Critias, who spoke vaguely of the "sickness of the country in consequence of the deforestation," and in 1540 by Fernando Colon, who declared that the rains in Maderia, the Azores, and the Canaries had become rarer since the trees had been cut down. But, in spite of these warnings, the process of "clearing" was carried on in most countries with reckless haste.

This havoc was not arrested until its consequences were pointed out by Humboldt, Boussingault and Becquerel, and by a still more authoritative teacher, experience, who, on this occasion, seems to have charged unusually high school fees. One of the most important effects of woods upon a climate is that they promote rain. The theory of this process is not perfectly understood, but the facts themselves are matters of experience. There are districts on the Continent where the chief rivers have decreased notably in volume since the clearing of the districts about their sources. We have seen a small stream, a tributary of the Oder, which, within the memory of living persons, turned in its course two or three corn mills. At the time of our visit it was dry all the summer months, save immediately after a thunder storm. In many districts of southern France the destruction of the forests has caused much more striking mischief.

The rain, instead of falling, as heretofore, in moderate showers, now comes in violent gushes, with long periods of drought between. As the natural consequence, the grasses and other low growing plants perish, their roots wither away, and the soil, no longer held together by their fibres, is washed away by the occasional violent rains and carried down into the beds of the rivers. The hillsides and the higher plains remain as barren wastes of sand, gravel and shingle. A similar process has been going on in Spain, Italy, Greece, Algeria, Morocco, and, in short, all around the Mediterranean. Countries which were once the granaries of the world, and which supported a numerous and thriving population, are now little better than deserts. Nor has this mischief been confined to Europe. The vegetable wealth of South Africa when it first became known to Europeans, was remarkable.

The Cape was the source of numbers of our finest greenhouse plants.

But now vast tracts have been rendered so desolate that a troop of the Colonial cavalry on the march actually gave three cheers at the sight of a tree. Even in the United States, once regarded as eminently the land of forests, many regions have lost, first, their vegetation and then their soil, in consequence of tree felling. It may, perhaps, here be objected that, fully admitting all these unfavorable changes, they may possibly have been produced by unknown causes, and would have occurred all the same if the woodlands had not been interfered with. This plea can easily be refuted. In many of the countries above mentioned replanting has been undertaken on a large scale by individuals, by communities and by Governments, and with the most decisive results.

Wherever such attempts have been made the climate becomes less extreme, the rainfall more uniformly distributed and public health improved. Such beneficial changes have been distinctly recognized in Northwestern India, where fertility is gradually returning to the deserts. In France, within about twenty years, 250,000 acres of mountain lands and nearly the same extent of sandy coast lands have been replanted—of course, at great expense, but with the most satisfactory results. In America, also, replanting is being vigorously carried on. An eminent agricultural authority in the United States has given it as his opinion that if one-fourth of a country is left covered with trees, the remaining three-fourths will yield a better return in the shape of crops than it would if stripped bare.—*Scientific News.*

